# When taking a step back is a veritable leap forward. Reversing decades of arthroscopy for managing joint pain: five reasons that could explain declining rates of common arthroscopic surgeries

**DOI:** 10.1136/bjsports-2020-102981

**Published:** 2020-10-16

**Authors:** Clare L Ardern, Teemu Paatela, Ville Mattila, Simo Taimela, Teppo L N Järvinen

**Affiliations:** 1 Sport & Exercise Medicine Research Centre, La Trobe University, Bundoora, Victoria, Australia; 2 Division of Physiotherapy, Karolinska Institute, Stockholm, Sweden; 3 Finnish Centre for Evidence-Based Orthopaedics (FICEBO), Department of Orthopaedics and Traumatology, University of Helsinki, Helsinki, Uusimaa, Finland; 4 Terveystalo Healthcare Services, Helsinki, Uusimaa, Finland; 5 Department of Orthopaedics and Traumatology, Tampere University Hospital, Tampere, Finland; 6 Department of Orthopaedics and Traumatology, Helsinki University Hospital, Helsinki, Finland

**Keywords:** arthroscopic surgery, evidence based, implementation, knowledge translation, overuse

Arthroscopy heralded an age of surgery-as-frontline-treatment for the painful joints of middle-aged and older people. By the end of the 20th century, knee, shoulder, hip and ankle arthroscopies were some of the most frequently performed surgeries in developed countries.^1^ Questions were first raised about the efficacy of knee arthroscopy for advanced osteoarthritis in 2002, when Professor Moseley and colleagues published their landmark placebo-controlled trial.^2^ Similarly, rigorous trials followed, each questioning the efficacy of arthroscopic partial meniscectomy and subacromial decompression—the two most common arthroscopic surgeries.

Despite compelling, high-quality evidence, why did the number of arthroscopies for degenerative conditions continue to rise in the first decade of the 21st century?^1^ The most obvious change to clinical practice was that arthroscopies were increasingly billed using different procedure (billing) codes.^3^ Our Finnish colleagues investigated the trends in various arthroscopic surgeries in Finland between 1997 and 2016 and found that the incidence of knee and shoulder arthroscopy peaked in 2006 and 2007, respectively, then steadily declined.^1^ The rates of wrist, elbow and hip arthroscopies also declined after their 2014 peak.^1^ Although the rates have declined in some countries, arthroscopies for patients with degenerative joint diseases remain some of the most commonly performed surgeries around the world.

The Finnish Centre for Evidence-Based Orthopaedics (FICEBO, www.ficebo.com) has been studying arthroscopic surgery for almost 20 years. During this period, we have observed dramatic changes in orthopaedics, debated why some orthopaedic surgeons continue to perform arthroscopy for degenerative musculoskeletal problems despite mounting evidence that arthroscopy is an ineffective procedure and discussed what might be driving other surgeons to change their practice.

Here, we explore five reasons that could explain reversals of arthroscopic surgery (medical reversal,^4^ based on our experience in Finland). This is not a definitive list—we encourage others in our community to share their thoughts and experiences.

## Reason 1: Healthcare funders have ceased reimbursing?

One might expect a policy issued by a government health authority—essentially banning arthroscopic surgery in Finland—would have the strongest impact on rates of surgery. However, the only formal statement issued by the Finnish government during the past decade is a recommendation by the Council for Choices in Health Care in Finland (COHERE). This permanent body, appointed by the Government, collaborates with the Ministry of Social Affairs and Health to issue recommendations on services that should be included in the range of public health services.

**Figure 1:**
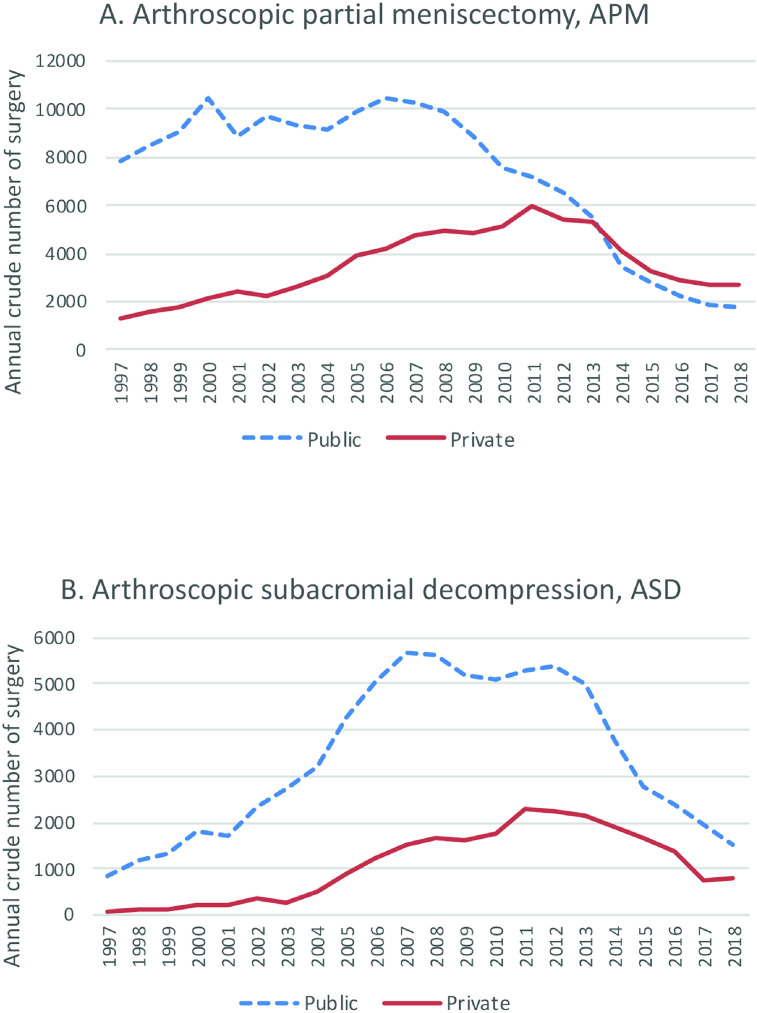
Annual number of arthroscopic partial meniscectomies for degenerative knee disease (A) and subacromial decompression of the shoulder (B) in the public (dotted line) and private (solid line) sector in Finland from 1997 to 2018.

In February 2017, COHERE declared that knee arthroscopy for degenerative knee disease should no longer be included in the range of public health services offered in Finland. However, the recommendation came almost a decade after the first surgeons stopped performing knee *and shoulder* arthroscopy. Rates of knee and shoulder arthroscopies in many European countries,^5^ and in the USA,^6^ have also steeply declined. To our knowledge, these medical reversals have also occurred without either the public or private healthcare sectors limiting reimbursement (figure 1).

## Reason 2: Professional arthroscopy (specialty) societies pulled the plug?

If one cannot congratulate the funders for limiting arthroscopy, perhaps a broad-based, international consensus on the need for medical reversal was responsible? One might expect such a consensus to be driven by medical specialty societies. However, many international arthroscopy societies have done exactly the opposite—they have published consensus statements and clinical practice guidelines that endorse knee and shoulder arthroscopy for middle-aged and older patients.

## Reason 3: A grass-roots movement overriding specialty societies?

**Figure 2:**
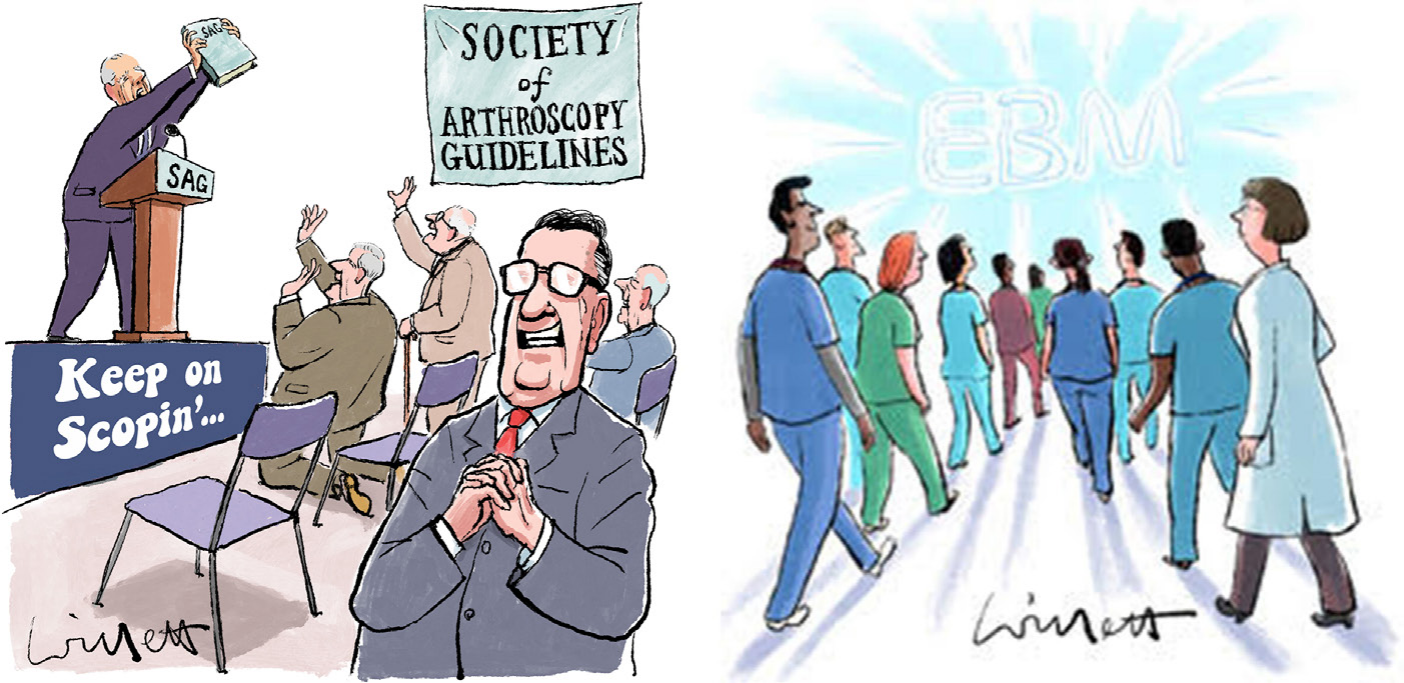
One artist’s impression of contemporary orthopaedic surgeons walking away from eminence-based treatment and now performing fewer arthoscopic surgeries (as per the trial evidence).

In response to specialty societies’ reluctance for change, we believe that individual surgeons are driving change—a ‘grass-roots’, clinician-led reversal of arthroscopy as first-line clinical practice.^7^ We know many orthopaedic surgeons who cannot understand why specialty societies continue to endorse arthroscopy. Guided by the evidence, these surgeons are abandoning arthroscopy *en masse* (figure 2)? And they are liberating their colleagues to do the same. Orthopaedic surgeons are rising to the challenge and responding responsibly to the evidence, despite financial incentives and the cognitive challenge of relinquishing cherished beliefs.

## Reason 4: General practitioners spearheading change?

The pivotal arthroscopy trials, published in general medical journals whose primary readership are general practitioners (GPs), suggest exercise therapy is a reasonable alternative to arthroscopy. We speculate that GPs have adopted the evidence: instead of indiscriminately referring middle-aged and older patients with knee or shoulder pain to orthopaedic surgeons, they may be steering patients to accessible and effective non-surgical management.^5 8^

## Reason 5: Consistent mass media messages have convinced patients of the futility of arthroscopy?

‘Useless surgery’ has been making the front page (https://www.nytimes.com/2016/08/04/upshot/the-right-to-know-that-an-operation-is-next-to-useless.html). One benefit of rigorous journalism is to help patients expand their knowledge of reasonable treatment options, including the harms and potential benefits of treatments. Knowledge empowers patients to participate in shared decision-making.

## Summary

Medical reversals—when clinicians stop performing ineffective procedures—are often gruelling and painstaking.^4^ Reversal of arthroscopy is happening—clinicians and patients are choosing wisely. As medical overuse has financial and health implications, the lessons from arthroscopy may be apposite to other medical interventions ripe for reversal.^4^

## References

[1] Karelson MC, Jokihaara J, Launonen AP, et al Lower nationwide rates of arthroscopic procedures in 2016 compared with 1997 (634925 total arthroscopic procedures): has the tide turned? Br J Sports Med 2020. 10.1136/bjsports-2019-101844. [Epub ahead of print: 02 Apr 2020]. PMC8408579 32241819

[2] Moseley JB, O'Malley K, Petersen NJ, et al A controlled trial of arthroscopic surgery for osteoarthritis of the knee. N Engl J Med 2002;347:81–8. 10.1056/NEJMoa013259 12110735

[3] Järvinen TLN, Guyatt GH Arthroscopic surgery for knee pain: a highly questionable practice without supporting evidence of even moderate quality. Br J Sports Med 2016;50:1426–7. 10.1136/bmj.i3934rep 30142083

[4] Prasad VK, Cifu AS Ending medical reversal : improving outcomes, saving lives. Baltimore: Johns Hopkins University Press, 2015.

[5] Siemieniuk RAC, Harris IA, Agoritsas T, et al Arthroscopic surgery for degenerative knee arthritis and meniscal tears: a clinical practice guideline. Br J Sports Med 2018;52:313. 10.1136/bjsports-2017-j1982rep 29449218 PMC5867409

[6] Howard DH Trends in the use of knee arthroscopy in adults. JAMA Intern Med 2018;178:1557–8. 10.1001/jamainternmed.2018.4175 30264154 PMC6584718

[7] Chen HY, Harris IA, Sutherland K, et al A controlled before-after study to evaluate the effect of a clinician led policy to reduce knee arthroscopy in NSW. BMC Musculoskelet Disord 2018;19:148. 10.1186/s12891-018-2043-5 29769120 PMC5956807

[8] Vandvik PO, Lähdeoja T, Ardern C, et al Subacromial decompression surgery for adults with shoulder pain: a clinical practice guideline. BMJ 2019;364:l294. 10.1136/bmj.l294 30728120

